# Incidence of preventable cardiopulmonary arrest in a mature part-time rapid response system: A prospective cohort study

**DOI:** 10.1371/journal.pone.0264272

**Published:** 2022-02-25

**Authors:** Myung Jin Song, Dong-Seon Lee, Yun-Young Choi, Da-Yun Lee, Hye-min Jo, Sung Yoon Lim, Jong Sun Park, Young-Jae Cho, Ho Il Yoon, Jae Ho Lee, Choon-Taek Lee, Yeon Joo Lee

**Affiliations:** 1 Division of Pulmonary and Critical Care Medicine, Department of Internal Medicine, Seoul National University College of Medicine, Seoul National University Bundang Hospital, Seongnam, South Korea; 2 Interdepartment of Critical Care Medicine, Seoul National University Bundang Hospital, Seongnam, Republic of Korea; Taipei Medical University School of Medicine, TAIWAN

## Abstract

**Background:**

The purpose of a rapid response system (RRS) is to reduce the incidence of preventable cardiopulmonary arrests (CPAs) and patient deterioration in general wards. The objective of this study is to investigate the incidence and temporal trends of preventable CPAs and determine factors associated with preventable CPAs in a hospital with a mature RRS.

**Methods:**

This was a single-center prospective cohort study of all CPAs occurring in the general ward between March 2017 and June 2020. The RRS operates from 07:00 to 23:00 on weekdays and from 07:00 to 12:00 on Saturdays. All CPAs were reviewed upon biweekly conference, and a panel of intensivists judged their preventability. Trends of preventable CPAs were analyzed using Poisson regression models and factors associated with preventable CPAs were analyzed using multivariable logistic regression.

**Results:**

There were 253 CPAs over 40 months, and 64 (25.3%) of these were preventable. The incidence rate of CPAs was 1.07 per 1000 admissions and that of preventable CPAs was 0.27 per 1000 admissions. The number of preventable CPAs decreased by 24% each year (incidence rate ratio = 0.76; p = 0.039) without a change in the total CPA incidence. The most common contributor to the preventability was delayed response from physicians (n = 41, 64.1%). A predictable CPA with a pre-alarm sign had increased odds in the occurrence of preventable CPAs, while a cardiac cause of CPAs and RRS operating hours had decreased odds in terms of occurrence of preventable CPA.

**Conclusion:**

Our study showed that one-fourth of all CPAs occurring in the general wards were preventable, and these arrests decreased each year. A mature RRS can evolve to reduce preventable CPAs with regular self-evaluation. Efforts should be directed at improving physicians’ response time since a delay in their response was the most common cause of preventable CPAs.

## Introduction

In-hospital cardiopulmonary arrest (CPA) is a highly morbid event with a mortality risk of approximately 80% [1]. Considering that patient populations and CPA definitions vary considerably between studies, the incidence rate of in-hospital cardiac arrest is reported to be 2–5 per 1,000 hospital admissions [1–3]. Hospital settings provide an opportunity to note a patient’s deterioration and hence anticipate a CPA, unlike out-of-hospital cardiac arrests. Previous studies consistently reported that more than two-thirds of in-hospital cardiac arrests were preceded by abnormal vital signs [2,4]. If these deteriorations are recognized and acted upon properly, in-hospital cardiac arrest can be prevented. A rapid response system (RRS) is an intervention to reduce potentially preventable CPAs by identifying at-risk patients and moving critical care resources to the bedside [5].

Our hospital implemented a part-time RRS in October 2012. Soon after implementing the RRS, we compared the numbers of CPAs before and after implementation and found that the part-time RRS reduced the total CPAs, which was attributable to a reduced CPA incidence rate during the RRS operating hours [6].

There is no standard definition of a “mature RRS”. However, the rapid response team (RRT) dose (RRT activations per 1,000 admissions) ranges from 25 to 55 has been suggested as an evaluation criterion for RRS maturity based on evidence of a dose-related reduction in CPA incidences [7–9]. Our hospital’s RRS meets the above criterion of a mature RRS.

Although RRS aims to prevent CPAs, and several studies [10,11], including the above-mentioned of ours, have shown RRS to reduce CPAs, it is impossible to prevent all CPAs. There are inevitable events that cannot be prevented. Therefore, the ultimate goal of RRS should be to reduce potentially preventable CPAs. From this aspect, evaluating the function of RRS with the incidence of total CPAs can underestimate the actual capacity of RRS. In order to evaluate the quality of RRS properly, it is necessary to measure potentially preventable CPAs.

Consequently, using a prospective RRS cohort data, we aimed to assess the incidence and temporal trends of preventable CPAs and evaluate factors associated therewith in a hospital with a mature RRS.

## Materials and methods

### Study design and population

We used the prospectively collected RRS data at Seoul National University Bundang Hospital, a 1,360-bed tertiary care hospital, that included data from adult patients (≥18 years old) who caused an RRS activation or underwent CPA in the general ward. We selected patients that underwent CPA between March 2017 and June 2020. CPA events that occurred in the emergency department, operating room, cardiac catheterization laboratory, or the intensive care unit (ICU) were not considered. All CPAs during the study period were reviewed at an RRS conference to assess their preventability. We evaluated the incidence and temporal trends of preventable CPAs and compared the characteristics of preventable and non-preventable CPAs to investigate the factors associated with occurrence of preventable CPAs.

This study was approved by the Institutional Review Board of the Seoul National University Bundang Hospital (protocol number: B-1604/344-106). Participant informed consent was waived because the research involved no more than minimal risk and was expected to improve patient safety. The study was conducted as per the principles expressed in the Declaration of Helsinki 1964 and its amendments.

### RRS at SNUBH

Our part-time RRS, named “*Seoul National University Bundang Hospital Medical Alert First Responder*” (i.e., SAFER), was implemented in October 2012. The RRT comprises 5 dedicated nurses and 16 dual-appointment physicians (internal medicine, anesthesiology, emergency medicine, and thoracic surgery). The RRS operates from 07:00 to 23:00 on weekdays and from 07:00 to 12:00 on Saturdays. During daytime hours (07:00 to 18:00 on weekdays), two physicians lead the RRT, each in charge of a medical and a surgical department. During the night (18:00 to 23:00 on weekdays) and on Saturdays (07:00 to 12:00), one physician leads the RRT. Two RRT nurses are always on duty during the RRS operating times.

Our team holds biweekly conferences to review all cases of patients who activated the RRS and had been transferred from the general ward to the ICU or experienced a CPA in the general ward in the preceding 14 days.

### Definition of predictable CPAs

Predictable CPAs are defined as any CPA occurring in hospitalized ward patients who meet the hospital’s escalation criteria at least 30 min before and within 24 h of the CPA, according to the statement on RRS evaluation by the International Society for Rapid Response Systems (ISRRS) [12].

Ten escalation criteria trigger the RRS in our hospital: a systolic blood pressure of <90 mmHg, heart rate of <50/min or >140/min, respiratory rate of <10/min or >30/min, a body temperature of >39°C or <36°C, peripheral oxygen saturation of <90% on room air or with a facial mask and oxygen flow of >8 L/min, a serum pH of <7.3, a partial pressure of carbon dioxide >50 mmHg or of oxygen <60 mmHg, serum lactic acid concentration of >2.5 mmol/L, and serum total carbon dioxide of <15 mmol/L.

If any of these criteria were observed before the CPA (within at least 30 min or maximally 24 h), then the case was defined as a “predictable CPA” with pre-alarm signs.

### Definition of preventable CPAs

The panel members were board-certified intensivists and met biweekly to assess the preventability of the CPAs that occurred over the past two weeks. The case reviews were performed as previously described by Hayward et al. [13], and the panel members were asked to answer the question, “Was the patient’s death preventable with better quality of care?” A preventable CPA was defined as one in which the panel members unanimously determined that it was preventable [14–16]. The median number of panel members attending each conference was 3.0 (interquartile range, 3.0–4.0).

The identified contributors for preventability were: a physician’s delayed response to a patient’s deterioration, failure to apply the patient care policy, drug errors or side effects, procedural errors, a delayed escalation from nurse to physician, and other causes closely associated with the CPA [16–18].

### Data collection

We collected the demographic information, comorbidities, return of spontaneous circulation, the Acute Physiology and Chronic Health Evaluation (APACHE) II score, the ICU stay length, the total hospital stay, and the hospital survival data of the prospectively enrolled patients from the electronic medical records. The total monthly admission volume and the case-mix index were retrieved from the hospital administrative data. The case-mix index is the average relative diagnosis-related group weight of a hospital’s inpatient discharges, reflecting the clinical complexity and resource needs of all patients in the hospital.

### Statistical analyses

The chi-square test was used to compare categorical variables, and the Student’s t-test was used to compare continuous variables. Poisson regression models were used to analyze the total CPA and preventable CPA incidence trends. Linear regression models were used to analyze the case-mix index and RRT dose trends. Factors associated with preventable CPA were assessed using multivariable logistic regression.

Statistical significance was defined as a p-value of less than 0.05. All data analyses were performed with R statistical software, version 4.0.0 (R Foundation, Vienna, Austria).

## Results

### Incidence of preventable CPAs and baseline characteristics based on preventability

There were 253 CPAs during the 40-month study period. The total CPA incidence was 1.07 per 1000 admissions. The mean age of CPA patients was 70.2 years, and 61.3% were male. Of the total CPA events, 64 cases (25.3%) were considered preventable. The preventable CPA incidence was 0.27 per 1000 admissions. Table 1 presents the baseline characteristics of CPA events based on preventability.

**Table 1:** Baseline characteristics of cardiopulmonary arrest based on preventability.

Variable		Total (n = 253)	Preventable (n = 64)	Unpreventable (n = 189)	p-value
Age		70.2 ± 11.7	70.0 ± 10.0	70.2 ± 12.3	0.881
Sex (Male)		155 (61.3%)	39 (60.9%)	116 (61.4%)	> 0.999
Charlson comorbidity score		3.0 ± 2.3	2.6 ± 2.2	3.1 ± 2.3	0.120
Affiliated department					0.183
	Surgical department	41 (16.2%)	7 (10.9%)	34 (18.0%)	
	Medical department	212 (83.8%)	57 (89.1%)	155 (82.0%)	
Arrest place					0.277
	General ward	220 (87.0%)	53 (82.8%)	167 (88.4%)	
	High dependency unit	22 (8.7%)	8 (12.5%)	14 (7.4%)	
	Hemodialysis room	7 (2.8%)	1 (1.6%)	6 (3.2%)	
	Diagnostic area	3 (1.2%)	1 (1.6%)	2 (1.1%)	
	Intra-hospital transporting	1 (0.4%)	1 (1.6%)	0 (0.0%)	
Cause of CPAs					< 0.001
	Cardiac	52 (20.6%)	2 (3.1%)	50 (26.5%)	
	Respiratory	67 (26.5%)	23 (35.9%)	44 (23.3%)	
	Aortic dissection	1 (0.4%)	0 (0.0%)	1 (0.5%)	
	Drug related	8 (3.2%)	3 (4.7%)	5 (2.6%)	
	Bleeding	33 (13.0%)	8 (12.5%)	25 (13.2%)	
	Metabolic	12 (4.7%)	5 (7.8%)	7 (3.7%)	
	Neurologic	13 (5.1%)	4 (6.2%)	9 (4.8%)	
	Sepsis	21 (8.3%)	11 (17.2%)	10 (5.3%)	
	Other	20 (7.9%)	1 (1.6%)	19 (10.1%)	
	Unknown	26 (10.3%)	7 (10.9%)	19 (10.1%)	
Predictable arrest with pre-alarm sign		90 (35.6%)	38 (59.4%)	52 (27.5%)	< 0.001
RRS operating time		117 (46.2%)	21 (32.8%)	96 (50.8%)	0.019
Outcomes					
	ROSC	154 (60.9%)	41 (64.1%)	113 (59.8%)	0.647
	ICU admission	141 (55.7%)	36 (56.2%)	105 (55.6%)	1.000
	Lengths of stay, hospital (day)	13.5 (6.0–26.25)	14.5 (9.0–35.0)	13.0(5.5–25.0)	0.204
	In-hospital mortality	206 (81.4%)	55 (85.9%)	151 (79.9%)	0.508
	CPC score 1 or 2 at discharge	20 (7.9%)	3 (4.7%)	17 (9.0%)	0.398
Variables of patients admitted to ICU					
	APACHE II score at ICU admission	36.9 ± 9.4	40.6 ± 8.8	35.7 ± 9.4	0.006
	Lengths of stay, ICU (day)	4.0 (2.0–9.0)	5.0 (1.5–11.5)	4.0 (2.0–8.0)	0.530

APACHE, Acute Physiologic Assessment and Chronic Health Evaluation; CPA, cardiopulmonary arrest; CPC, Cerebral Performance Category; ICU, intensive care unit; ROSC, return of spontaneous circulation; RRS, rapid response system.

The distribution of CPA causes significantly differed based on preventability (p < 0.001). The most common preventable CPA cause was respiratory (35.9%), followed by sepsis (17.2%), whereas the most common unpreventable CPA cause was cardiac (26.5%), followed by respiratory (23.3%). A predictable CPA with a pre-alarm sign was observed in 90 of 253 CPAs (35.6%), with a significant difference between preventable and unpreventable CPAs (59.4% vs. 27.5%; p < 0.001).

The return of spontaneous circulation, ICU admission, the length of hospital stay, and in-hospital mortality did not differ based on the preventability. The APACHE II score of patients at ICU admission was significantly higher in those with preventable CPAs than in those with unpreventable CPAs, but the length of the ICU stay did not differ.

### CPA incidence trends

**Fig 1:**
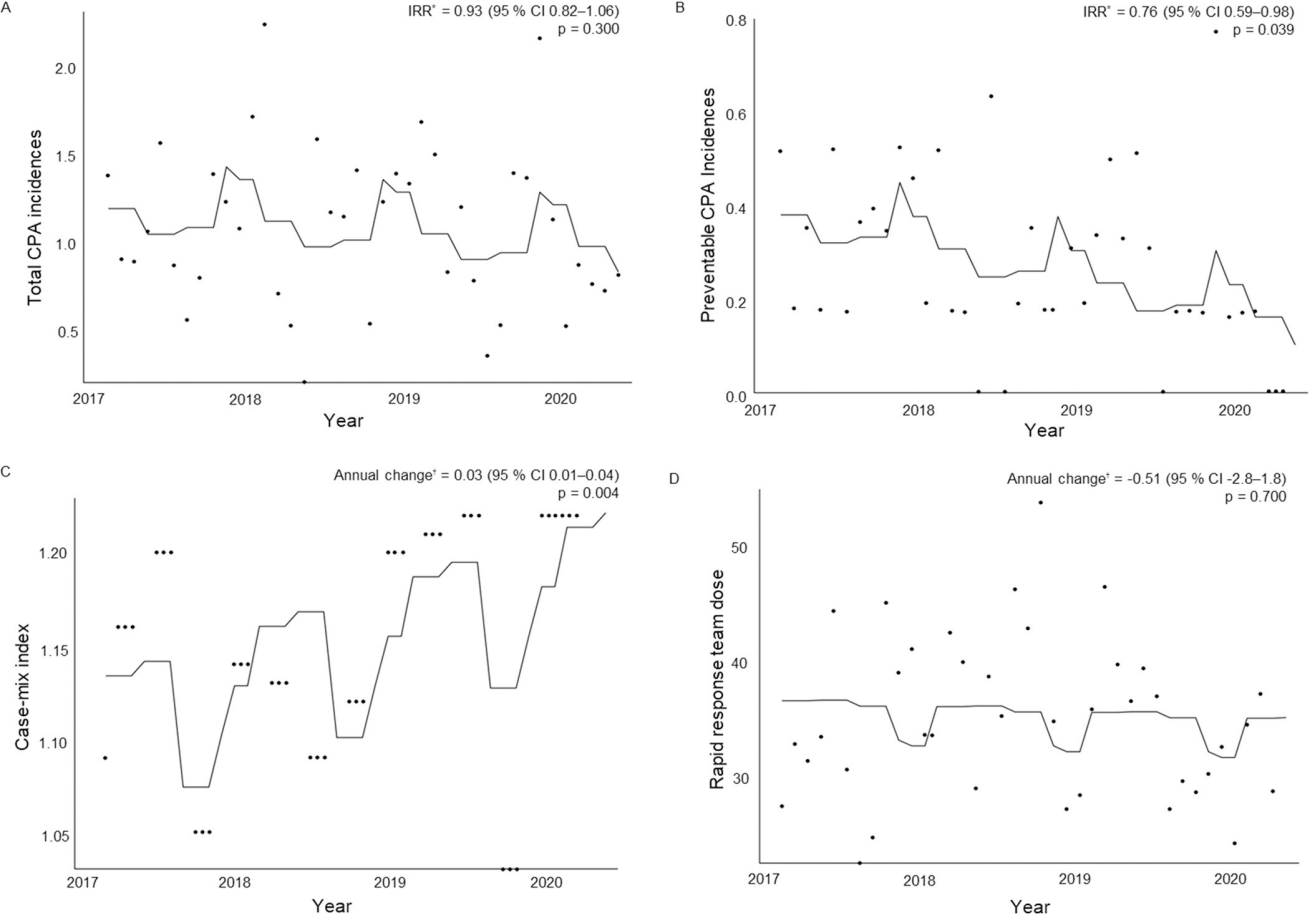
Annual cardiopulmonary arrest (CPA) incidence, case-mix index, and rapid response team dose trends. (a) Total CPA incidences, (b) preventable CPA incidences, (c) case-mix index, and (d) rapid response team dose (i.e., rapid response system activation per 1000 admissions). IRR: Incidence rate ratio, CI: confidence interval. *Estimated by the Poisson regression model. ^†^Estimated by a linear regression model.

Fig 1 present the annual trends of CPA incidence, case-mix index, and RRT dose. Analyses were carried out after adjusting for seasonality, considering seasonal variations in CPA incidence [19]. The overall total CPA annual incidence rate did not show a statistically significant difference during the study period (incidence rate ratio [IRR] = 0.93; 95% confidence interval [CI], 0.82–1.06; p = 0.300). However, the incidence of preventable CPAs significantly decreased by 24% each year during the study period (IRR = 0.76; 95% CI, 0.59–0.98; p = 0.039).

Fig 1C and 1D present the case-mix index and the RRT dose trends. The case-mix index statistically significantly increased by 0.03 points each year (β coefficient 0.03; 95% CI, 0.01–0.04; p = 0.004). The RRT dose did not change, averaging 34.93 ± 6.95 throughout the study period (β coefficient 0.51; 95% CI, -2.8–1.8; p = 0.700).

### CPAs during RRS operating and non-operating hours

**Fig 2:**
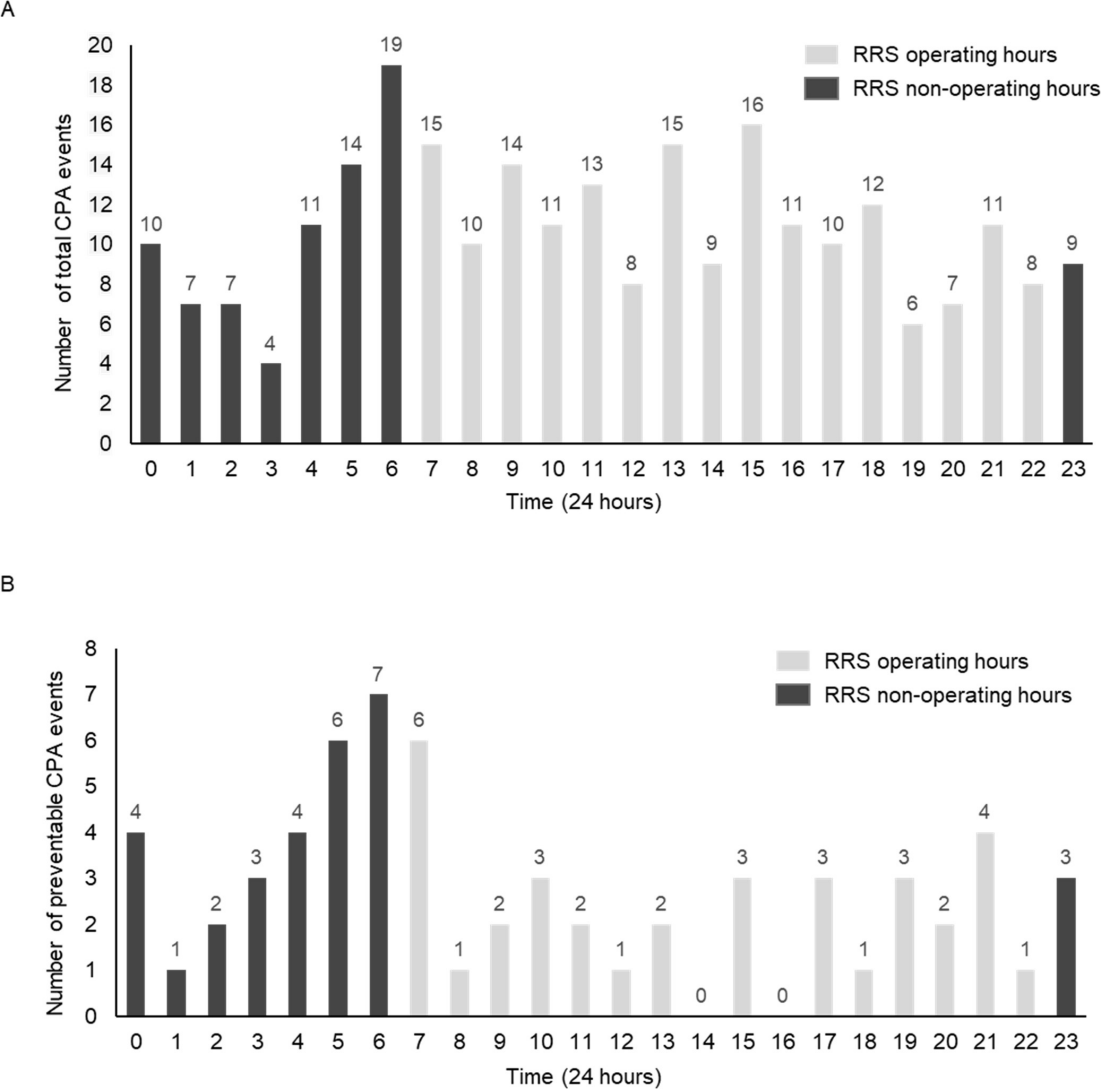
The number of cardiopulmonary arrests over 24 hours. (a) Total cardiopulmonary arrests; (b) Preventable cardiopulmonary arrests. RRS: rapid response system, CPA: cardiopulmonary event.

There were 117 (46.2%) CPAs during the RRS operating hours and 136 (53.8%) during non-operating hours (S1 Table). Preventable CPAs were significantly lower during the RRS operating hours than during the non-operating hours (17.9% vs. 31.6%; p = 0.019). Fig 2 shows the total number of CPAs over 24 h. The total numbers of CPAs were relatively evenly distributed over the 24-h period, whereas the number of preventable CPA events was low during the RRS operating hours and relatively higher during the non-operating hours.

### Contributors to the preventability

The most frequent contributor to the preventability was physicians’ delayed response to a deterioration of patients (n = 41, 64.1%). Failure to apply patient-care policies was the second most frequent contributor (e.g., patients with CPA caused by hemorrhagic shock who would have been eligible for blood product transfusion based on the transfusion guidelines but were not transfused; patients whose CPA was due to embolism and who would have been eligible but did not receive deep vein thrombosis prophylaxis; n = 15, 23.4%). Drug errors or side effects (n = 3, 4.7%), procedural errors (n = 3, 4.7%), and delayed escalation from nurse to physician (n = 1, 1.6%) were also noted. The contributors to the preventability did not differ between RRS operating and non-operating hours (Table 2).

**Table 2:** The contributors to the preventability defined by expert panel.

Reasons	Total (n = 64)	Operating hours (n = 21)	Non-operating hours (n = 43)	p- value
Doctors’ delayed response to deterioration	41 (64.1%)	12 (57.1%)	29 (67.4%)	0.503
Failure to practice established patients care policy	15 (23.4%)	5 (23.8%)	10 (23.3%)	
Drug error or side effect	3 (4.7%)	1 (4.8%)	2 (4.7%)	
Procedural errors	3 (4.7%)	2 (9.5%)	1 (2.3%)	
Delayed escalation from nurse to doctor	1 (1.6%)	1 (4.8%)	0 (0.0%)	
Other	1 (1.6%)	0 (0.0%)	1 (2.3%)	

### Factors associated with preventable CPA

A predictable arrest with a pre-alarm sign (OR = 3.19; 95% CI, 1.64–6.20; p < 0.001) had increased odds in occurrences of preventable CPAs. A causative cardiac event (adjusted odds ratio [OR] = 0.07; 95% CI, 0.01–0.25; p < 0.001) and the RRS operating hours (adjusted OR = 0.39; 95% CI, 0.19–0.78; p = 0.010) had decreased odds in terms of occurrence of preventable CPAs (Table 3).

**Table 3:** Logistic regression analysis for preventable cardiopulmonary arrest.

Variables	Univariable analysis			Multivariable analysis		
	OR	95% CI	p-value	Adjusted OR	95% CI	p-value
Charlson comorbidity score	0.90	0.79–1.03	0.121	0.87	0.74–1.01	0.065
Age	0.99	0.97–1.02	0.881	0.99	0.96–1.02	0.528
Sex	1.02	0.57–1.81	0.950	1.04	0.53–2.06	0.902
Cardiac cause	0.09	0.01–0.30	0.001	0.07	0.01–0.25	<0.001
Predictable arrest with pre-alarm sign	3.85	2.14–7.02	<0.001	3.19	1.67–6.20	<0.001
RRS operation	0.32	0.16–0.62	0.001	0.31	0.14–0.63	0.002

APACHE, Acute Physiologic Assessment and Chronic Health Evaluation; CPA, cardiopulmonary arrest; CPC, Cerebral Performance Category; ICU, intensive care unit; ROSC, return of spontaneous circulation; RRS, rapid response system.

## Discussion

This 40-month prospective cohort study analyzed CPAs at our hospital with a mature part-time RRS. We determined that the total CPA incidence was 1.07 per 1,000 admissions, and the preventable CPA incidence was 0.27 per 1000 admissions. During the study period, the incidence of preventable CPAs decreased by 24% annually without a change in the total CPA incidence and despite an increasing severity and complexity of patients’ conditions and steady RRT doses. A predictable CPA with a pre-alarm sign was at increased odds in occurrence of preventable CPAs, while a cardiac cause of CPAs and RRS operating hours were at decreased odds in occurrence of preventable CPAs.

The overall CPA incidence rate is a quality control RRS metric widely used in studies. However, this only represents a crude proxy for the incidence of preventable CPAs, even though preventable CPA prevention is the ultimate goal of an RRS. Moreover, the effectiveness of an RRS can be obscured by including “inevitable” CPAs in the overall incidence rate. This is especially true in the case of mature RRS, which the incidence of total CPA is lower than that reported in hospitals with only recently implemented RRSs [11]. Several studies have evaluated preventable CPAs directly, albeit with variable definitions of a preventable CPA. These reports indicate that approximately 14–27% of CPA events are preventable, and a similar percentage of preventable CPAs are recorded even in institutions operating a mature RRS [16,17,20–22].

Our study showed that preventable CPAs decreased with continuous quality improvement of the RRS, through self-evaluation and feedback process, despite the total CPA incidence rate already being low, which is a novel result. During the 40-month study period, the incidence rate of preventable CPAs decreased each year without changes in the total CPA incidence rate. This is a notable result considering that the case-mix index increased, while the RRT doses did not change each year, satisfying the criterion for the mature RRS. This result indicates that even though the complexity and severity of patients’ conditions increased, the RRT acted effectively at a steady dose and reduced the incidence of preventable CPAs.

The quality control method of the RRS at our hospital is as follows. An RRS conference was held at our hospital every two weeks to review the CPA events in the general ward and all cases transferred from the general ward to the ICU. The RRT members and the staff in charge of the ICU participated in the conference. The steps to potentially preventable CPAs were explored according to the study protocol, and the management in the general ward and the patient’s progress after ICU transfer were also shared to provide feedback on the quality of the RRT’s patient care. This thorough feedback process allowed the RRT to progress, resulting in better patient outcomes.

The most common contributor to the preventability of CPAs was the physician’s delayed response. Several studies have shown that delayed evaluation by the RRT and critical care intervention increase patient morbidity and mortality [23–25]. Additional information on the timeliness of the response might help reduce the number of delayed responses. The ISRRS recommends two evaluation metrics: the timeliness of the RRT decision-maker’s initial evaluation (from escalation criteria breach to the initial evaluation) and the timeliness of critical care interventions (from escalation criteria breach to the critical care intervention) [12]. The ISRRS suggests that a critical care intervention should occur within 6 h based on previous retrospective studies [23–27]. Further studies are needed to evaluate if assessing the timeliness of the response can actually reduce delayed responses and decrease preventable CPAs.

Although the incidence of preventable CPA was lower during the RRS operating time compared with the non-operating time, there was no difference in the outcome of the CPAs that occurred, regardless of RRS operating time. In the case of our hospital, cardiopulmonary resuscitation (CPR) is leaded by CPR leader (apart from the RRT) and the management after ROSC is performed in the ICU. These policies of our hospital might be associated with why the results were what they were, that there is no difference in the outcome of CPA that were already about to occur. However, reducing the incidence of preventable CPAs is definitely beneficial in terms of patient safety.

Our study has several limitations. First, determining CPA preventability can be subjective and shows discrepancies in decisions by the panel. Previous studies aiming to assess preventable mortality have also struggled with this issue [28,29]. However, we mitigated the subjectivity by limiting the panel members to experienced intensivists, using case review training sets and a structured medical record review. Second, since we reviewed the electronic medical records to define CPA preventability, we could not assess details of the actual CPA situation that were not recorded. Third, the single-center design of this study limits the generalizability to other hospital systems.

## Conclusions

The mature part-time RRS in this study showed that one-fourth of all CPAs occurring in general wards were preventable. Furthermore, the preventable CPA incidence decreased each year. This study shows that even a mature RRS with a low overall CPA incidence rate can evolve and improve patient safety further by regular self-evaluation based on feedback. Future efforts should be directed at improving the physician’s response time.

## Supporting information

S1 Table The baseline characteristics of patients based on the rapid response system operating housrs. (DOCX)

S2 Table List of individual cardiopulmonary arrest cases. (XLSX)

S3 Table List of monthly cardiopulmonary arrest cases. (XLSX)

## Abbreviations

RRS: Rapid response system
CPAs: Cardiopulmonary arrests
RRT: Rapid response team
ICU: Intensive care unit
ISRRS: International Society for Rapid Response Systems
APACHE: Acute Physiology and Chronic Health Evaluation
IRR: Incidence rate ratio
OR: Odds ratio
