# Supplementary material for: Incidence of preventable cardiopulmonary arrest in a mature part-time rapid response system: A prospective cohort study
Source: PLoS One. 2022 Feb 25;17(2):e0264272. doi: 10.1371/journal.pone.0264272 (PMC8880884; doi:10.1371/journal.pone.0264272)
Supplement: S1 Table — (DOCX) [file pone.0264272.s001.docx]

**Table S1.** The baseline characteristics of patients based on the rapid response system operating hours

|  |  | Operating hours  (n = 117) | Non-operating hours  (n = 136) | p-value |
| --- | --- | --- | --- | --- |
| Age | | 70.9±11.5 | 69.5±11.9 | 0.370 |
| Sex (Male) | | 70 (59.8%) | 85 (62.5%) | 0.760 |
| Charlson Comorbidity Score | | 3.0±2.4 | 3.0±2.2 | 0.786 |
| Affiliated department | |  |  | 0.900 |
|  | Surgical department | 20 (17.1%) | 21 (15.4%) |  |
|  | Medical department | 97 (82.9%) | 115 (84.6%) |  |
| Location of CPA | |  |  | 0.045 |
|  | General ward | 96 (82.1%) | 124 (91.2%) |  |
|  | High dependency unit | 11 (9.4%) | 11 (8.1%) |  |
|  | Hemodialysis room | 6 (5.1%) | 1 (0.7%) |  |
|  | Diagnostic area | 3 (2.6%) | 0 (0.0%) |  |
|  | Intra-hospital transporting | 1 (0.9%) | 0 (0.0%) |  |
| Cause of CPA | |  |  | 0.036 |
|  | Cardiac | 24 (20.5%) | 28 (20.6%) |  |
|  | Respiratory | 42 (35.9%) | 25 (18.4%) |  |
|  | Aortic dissection | 1 (0.9%) | 0 (0.0%) |  |
|  | Drug-related | 2 (1.7%) | 6 (4.4%) |  |
|  | Bleeding | 15 (12.8%) | 18 (13.2%) |  |
|  | Metabolic | 6 (5.1%) | 6 (4.4%) |  |
|  | Neurologic | 4 (3.4%) | 9 (6.6%) |  |
|  | Sepsis | 4 (3.4%) | 17 (12.5%) |  |
|  | Other | 8 (6.8%) | 12 (8.8%) |  |
|  | Unknown | 11 (9.4%) | 15 (11.0%) |  |
| Predictable arrest with pre-alarm sign | | 26 (22.2%) | 64 (47.1%) | < 0.001 |
| Preventable CPA | | 21 (17.9%) | 43 (31.6%) | 0.019 |
| Outcome | |  |  |  |
|  | ROSC | 72 (61.5%) | 82 (60.3%) | 0.942 |
|  | ICU admission | 69 (59.0%) | 72 (52.9%) | 0.403 |
|  | Lengths of stay, hospital (day) | 37.2 ± 111.3 | 21.4 ± 38.7 | 0.123 |
|  | In-hospital mortality | 91 (77.8%) | 115 (84.6%) | 0.255 |
|  | CPC score 1 or 2 at discharge | 7 (6.0%) | 13 (9.6%) | 0.425 |
| Variables of patients admitted to ICU | | |  |  |
|  | APACHE II score at ICU admission | 36.7 ± 9.0 | 37.2 ± 9.9 | 0.741 |
|  | Lengths of stay, ICU (day) | 19.1 ± 104.7 | 6.4 ± 6.3 | 0.308 |

APACHE, Acute Physiologic Assessment and Chronic Health Evaluation; CPA, cardiopulmonary arrest; CPC, Cerebral Performance Category; ICU, intensive care unit; ROSC, return of spontaneous circulation; RRS, rapid response system
